# Genomic profiling of drug resistance markers in *Plasmodium falciparum* samples from the Democratic Republic of the Congo (2017) and Tanzania (2020–2021)

**DOI:** 10.1093/jac/dkag309

**Published:** 2026-09-01

**Authors:** Flory Luzolo Khote, Hypolite Muhindo Mavoko, Patrick Mitashi, Papy Mandoko, Destin Mbongi, Geofrey Makenga, Daniel T R Minja, Filbert Francis, Reginald Kavishe, Jean-Pierre van Geertruyden, Gauthier Mesia Kahunu, Didier Menard, Vito Baraka

**Affiliations:** Department of Tropical Medicine, University of Kinshasa, Kinshasa, Democratic Republic of the Congo; Global Health Institute, University of Antwerp, Antwerp, Belgium; Department of Tropical Medicine, University of Kinshasa, Kinshasa, Democratic Republic of the Congo; Department of Tropical Medicine, University of Kinshasa, Kinshasa, Democratic Republic of the Congo; Departement de parasitologie, Institut National des Recherches Biomédicales (INRB), Kinshasa, Republique Démocratique du Congo; Centre Hospitalier Monkole, Kinshasa, Democratic Republic of the Congo; Global Health Institute, University of Antwerp, Antwerp, Belgium; National Institute for Medical Research, Tanga, Tanzania; National Institute for Medical Research, Tanga, Tanzania; National Institute for Medical Research, Tanga, Tanzania; Kilimanjaro Clinical Research Institute (KCRI), Kilimanjaro Christian Medical University College (KCMUCo), Tumaini University Makumira, Moshi, Tanzania; Global Health Institute, University of Antwerp, Antwerp, Belgium; Department of Pharmacology and Therapeutics, University of Kinshasa, Kinshasa, Democratic Republic of the Congo; Malaria Genetics and Resistance Team (MEGATEAM), UR 3073—Pathogens Host Arthropods Vectors Interactions Unit, Université de Strasbourg, Strasbourg F-67000, France; Malaria Parasite Biology and Vaccines, INSERM Unit 1347—ParasitInnov, Institut Pasteur, Université Paris Cité, Paris F-75015, France; Laboratory of Parasitology and Medical Mycology, CHU Strasbourg, Strasbourg F-67000, France; Institut Universitaire de France (IUF), Paris F-75231, France; National Institute for Medical Research, Tanga, Tanzania

## Abstract

**Background:**

Molecular surveillance is essential to detect emerging artemisinin partial resistance (ART-R) and partner drug resistance in Sub-Saharan Africa.

**Objectives:**

To describe the prevalence of *Plasmodium falciparum* resistance markers in two high-burden countries using artesunate-amodiaquine (ASAQ) and artemether-lumefantrine (AL): the Democratic Republic of the Congo (DRC) and Tanzania.

**Methods:**

A total of 1254 day-0 *P. falciparum*-positive samples were analysed: 837 from four sentinel sites of a therapeutic efficacy study (TES) in the DRC (2017) and 417 from an intermittent preventive treatment in schoolchildren (IPTsc) trial in Handeni and Kilindi districts, Tanga Region, Tanzania (2020–2021). *Pfkelch13*, *Pfcrt* and *Pfmdr1* were analysed by Illumina MiSeq amplicon sequencing.

**Results:**

Reliable sequences were obtained for 1193 isolates. The *Pfkelch13* wild-type allele predominated (98.4%); none of the four non-synonymous mutations detected (N489Y, K568T, A578S, V589I) are classified as validated, candidate or potential ART-R markers, and the validated markers R561H, P441L and C469Y reported elsewhere in East Africa were absent. *Pfcrt* K76T was found in 23.3% of Tanzanian and 26.3% of DRC isolates, with substantial between-site variation in the DRC (4.3% to 93.6% at Rutshuru). *Pfmdr1* haplotype profiles differed between countries: N**F**SND predominated in Tanzania (71.5%) while NYSND remained the most frequent in the DRC (60.9%); 86Y was twice as frequent in the DRC (11.1%) as in Tanzania (6.2%).

**Conclusions:**

No validated ART-R marker was detected, but partner-drug haplotype distributions reflected the first-line ACTs used in each country. Continued molecular surveillance is needed to track these signatures alongside the recent emergence of ART-R.

## Introduction

Sub-Saharan Africa (SSA) bears the highest global malaria burden, accounting for more than 90% of malaria-related deaths, with children under five years of age remaining the most affected group.^1^  *Plasmodium falciparum (P. falciparum)*, the dominant malaria species in SSA, has repeatedly developed resistance to successive first-line antimalarial drugs.^2,3^

Artemisinin-based combination therapies (ACTs) are the WHO-recommended first-line treatment for uncomplicated *P. falciparum* malaria and have substantially reduced malaria morbidity and mortality over the past two decades.^4^ However, the emergence and spread of ART-R, characterized by delayed parasite clearance following treatment, threatens these gains.

First reported in Southeast Asia in 2008,^5^ ART-R has since emerged independently in several African countries, including Rwanda,^6,7^ Uganda,^8^ Eritrea^9^ and Tanzania.^10–12^ WHO currently recognizes fourteen *Pfkelch13* mutations as validated ART-R markers, including R561H, C469Y, A675 V, R622I and C580Y.^13,14^

Resistance to ACT partner drugs may further compromise treatment efficacy. The *Pfcrt* K76T mutation is a validated marker of chloroquine (CQ) resistance,^15,16^ while only the extended *Pfcrt* 7G8 haplotype (C72S + K76T + A220S + N326D + I356L) and the *Pfmdr1* N86Y mutation have been listed as candidate markers for reduced susceptibility to amodiaquine (AQ).^14,17^ To date, no validated or candidate marker of lumefantrine resistance has been established.^14,18^

The DRC and Tanzania remain among the countries with the highest malaria burden worldwide and rely extensively on ACTs. Tanzania has reported confirmed ART-R,^12^ while validated and candidate *Pfkelch13* resistance mutations have recently been detected in the DRC, which shares borders with Uganda, Rwanda and Tanzania, all confirmed ART-R hotspot countries.^19,20^ Nevertheless, baseline data from both countries remain limited.

We assessed the prevalence of molecular markers of artemisinin and partner drug resistance in *P. falciparum* isolates collected in the DRC in 2017^21^ and in Tanzania between 2020 and 2021.^22^

## Methods

### Study sites and participants

Molecular markers of ART-R and partner drug resistance were assessed in *P. falciparum* positive samples collected from the DRC and Tanzania.

*DRC*: samples were collected in 2017 during a TES conducted at four National Malaria Control Programme (NMCP) sentinel sites.^21^ Kabondo (Tshopo province, north) and Mikalayi (Kasai-Central province, centre) are located in the equatorial zone with malaria rapid diagnostic test (mRDT) positivity of 52.2% and 45.5% in children aged 6–59 months, respectively. Rutshuru (North Kivu province, east), a mountainous zone, had an mRDT positivity rate of 11.4%. Kapolowe (Haut-Katanga province, south), in a tropical zone, had an mRDT positivity rate of 42.7%. Children aged 6–59 months with microscopically confirmed uncomplicated *P. falciparum* mono-infection were enrolled in health facilities according to the WHO TES protocol,^23^ with 178 samples per site.

*Tanzania*: Samples were collected from Schoolchildren aged 5–15 years attending primary school between July 2020 and December 2021 in Handeni and Kilindi districts (Tanga Region, northeastern Tanzania). A total of 4032 participants from both IPTsc intervention and control groups were screened at baseline.^22^ Among 417 *P. falciparum*-positive participants, 356 qPCR- confirmed samples with sufficient parasite DNA were retained for sequencing of antimalarial drug resistance markers. Written informed consent was obtained from parents or guardians, and assent was required for children aged ≥ 11 years.

### Study procedure

At enrolment, thin and thick blood smears were prepared to confirm parasite species, *P. falciparum* mono-infection, and parasite density. In the DRC, eligibility criteria followed the WHO TES protocol,^23^ including age 6–59 months, uncomplicated malaria with parasite density between 2000 and 200 000 parasites/μL, axillary temperature ≥ 37.5°C or recent history of fever, no recent antimalarial use, and absence of severe malaria or comorbidities. In Tanzania, schoolchildren were recruited from selected wards under IPTsc trial procedures.^22^ Capillary blood was collected before any antimalarial treatment and spotted onto Whatman 3MM filter paper for dried blood spot (DBS) storage.

### DNA extraction and molecular analysis

Parasite DNA was extracted from day 0 DBS using the QIAamp DNA Blood Mini Kit (Qiagen, Germany) according to the manufacturer's instructions. DNA was eluted in 100 μL buffer and stored at −20°C. Concentration was measured using a NanoDrop 1000 spectrophotometer (Thermo Fisher Scientific).

Molecular markers were analysed in three genes: the *Pfkelch13* propeller domain (codons 430–720), *Pfcrt* (codons 72–76, 93, 97, 145, 218, 343, 350, 353, 356), and *Pfmdr1* (codons 86, 184, 1034, 1042, 1246), associated with reduced susceptibility to artemisinin derivatives, 4-aminoquinolines and amino alcohols, respectively.

Targeted regions were amplified and sequenced as previously described.^9^ Briefly, multiplexed nested PCR assays with indexed primers carrying unique barcodes were used to generate amplicons. Pooled amplicons were purified using AMPure XP beads (Beckman Coulter), and library quality was assessed using a Fragment Analyzer (Agilent). Libraries were quantified fluorometrically (Qubit, Thermo Fisher Scientific), pooled, denatured with NaOH, and sequenced on an Illumina MiSeq platform (v2, 300-cycle kit).

Raw sequences were demultiplexed and quality-trimmed at a Phred score of 30. Primer sequences were trimmed to avoid primer bias. Reads were aligned to the *P. falciparum* 3D7 reference genome (v45) using CLC Genomics Workbench v25 (Qiagen). Mixed infections were defined using a minimum read depth of 20 and a minor allele frequency threshold of 5%. Reference laboratory strains (Dd2, 7G8, HB3, and Cambodian strain 3601 carrying *Pfkelch13* C580Y) were included as controls. The 3601 strain served as a positive control for *Pfkelch13* C580Y mutation detection, whereas Dd2, 7G8, and HB3 provided reference controls for *Pfcrt* and *Pfmdr1* polymorphism detection and sequencing validation.

### Statistical analysis

Analyses were performed using MedCalc v23.5 (MedCalc Software Ltd, Ostend, Belgium) and GraphPad Prism v11 (GraphPad Software, Boston, MA, USA). SNP and haplotype prevalences were summarized as proportions with 95% confidence intervals (Clopper-Pearson method). Results were reported separately by country and site. No statistical comparison between countries was performed due to differences in study design, sampling periods, and age groups.

Only samples passing gene-specific sequencing quality thresholds were included in each analysis; samples failing one gene were retained for others. Mixed alleles (wild-type/mutant) were classified as mutant. Haplotypes for *Pfcrt* (codons 72–76) and *Pfmdr1* were inferred directly from sequencing reads, and samples with missing codons were excluded from haplotype analyses. Spatial distribution of resistance markers was visualized using Magrit (https://magrit.cnrs.fr/). Categorical variables were compared using Pearson’s chi-square test or Fisher’s exact test when appropriate. A *P*-value < 0.05 was considered statistically significant.

Ethical approval. In the DRC, ethical approval was obtained from the Ethics Committee of the School of Public Health, University of Kinshasa, and the study was registered (ClinicalTrials.gov: NCT02940756).^21^ In Tanzania, approvals were obtained from the Medical Research Coordinating Committee (NIMR/HQ/R.8a/Vol.IX/3291 and extension NIMR/HQ/R.8c/Vol.I/1652) and the Tanzania Medicines and Medical Devices Authority (TMDA0019/CTR/0018/03).^22^ The study adhered to the Declaration of Helsinki and national regulations. Written informed consent was obtained from parents or legal guardians, and assent from children aged ≥11 years.

## Results

### Sample collection and sequencing outcomes

**Figure 1.**
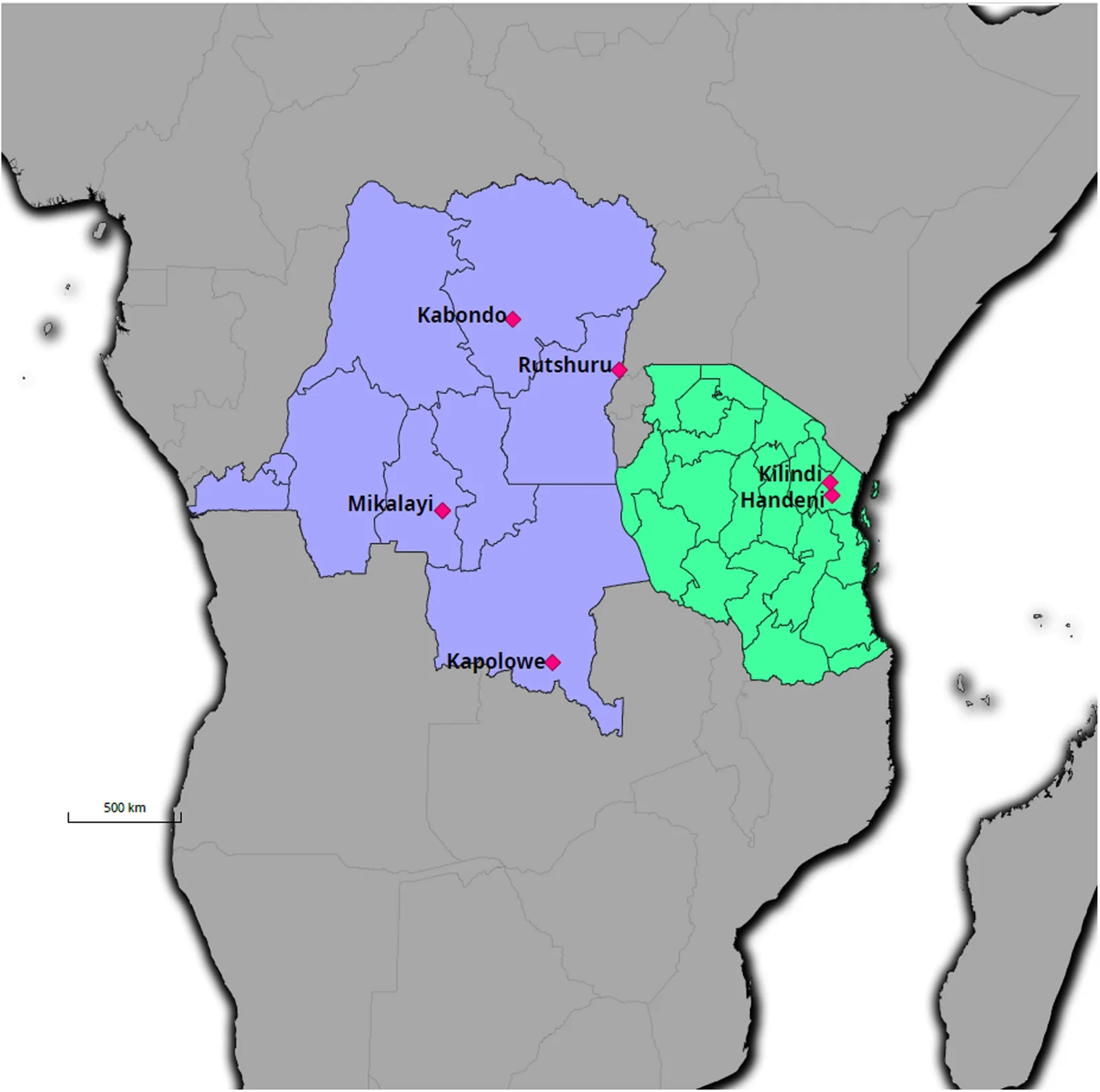
Geographical location of the study sites. Four sentinel sites of the National Malaria Control Programme of the Democratic Republic of the Congo (Kabondo, Kapolowe, Mikalayi, Rutshuru) where samples were collected during a therapeutic efficacy study in 2017, and two districts of the Tanga Region in Tanzania (Handeni, Kilindi) where samples were collected during an intermittent preventive treatment in school children (IPTsc) trial between July 2020 and December 2021. Map produced using Magrit (https://magrit.cnrs.fr).

A total of 1254 *P. falciparum*-positive samples were collected: 417 from Tanzania (Handeni, *n* = 268; Kilindi, *n* = 149; 2020–2021) and 837 from the DRC (Kabondo, *n* = 236; Kapolowe, *n* = 185; Mikalayi, *n* = 259; Rutshuru, *n* = 157; 2017). The geographical distribution of study sites is shown in Figure 1. After sequencing and quality filtering, 356/417 (85.4%) Tanzanian and 837/837 (100%) DRC samples yielded reliable sequences for *Pfkelch13*, *Pfcrt* and *Pfmdr1*. Sequencing success was similar between Tanzanian sites (Handeni 84.0%, Kilindi 87.9%; *P* = 0.301, Fisher’s exact test; Table 1).

**Table 1.** Sequencing success and prevalence of single nucleotide polymorphisms in *Pfkelch13*, *Pfcrt* and *Pfmdr1* genes per study site, in Tanzania (2020–2021) and the Democratic Republic of the Congo (2017)

Gene	Codon	AA	TANZANIA				DRC					
			Handeni	Kilindi	Total	*P*-value*	Kabondo	Kapolowe	Mikalayi	Rutshuru	Total	*P*-value*
No. of samples sequenced			225	131	356		236	185	259	157	837	
*Pfkelch13*	WT	—	225 (100.0%)	115 (87.8%)	340 (95.5%)	**<0**.**001**	234 (99.2%)	184 (99.5%)	259 (100.0%)	157 (100.0%)	834 (99.6%)	0.355
	489	N > Y	0 (0.0%)	4 (3.1%)	4 (1.1%)	**0**.**018**	0 (0.0%)	0 (0.0%)	0 (0.0%)	0 (0.0%)	0 (0.0%)	—
	568	K > T	0 (0.0%)	12 (9.2%)	12 (3.4%)	**<0**.**001**	0 (0.0%)	0 (0.0%)	0 (0.0%)	0 (0.0%)	0 (0.0%)	—
	578	A > S	0 (0.0%)	1 (0.8%)	1 (0.3%)	0.368	2 (0.8%)	0 (0.0%)	0 (0.0%)	0 (0.0%)	2 (0.2%)	0.164
	589	V > I	0 (0.0%)	0 (0.0%)	0 (0.0%)	—	0 (0.0%)	1 (0.5%)	0 (0.0%)	0 (0.0%)	1 (0.1%)	0.317
*Pfcrt*	72	C	215 (95.6%)	130 (99.2%)	345 (96.9%)	0.061	235 (99.6%)	185 (100.0%)	258 (99.6%)	155 (98.7%)	833 (99.5%)	0.385
		S	10 (4.4%)	1 (0.8%)	11 (3.1%)		1 (0.4%)	0 (0.0%)	1 (0.4%)	2 (1.3%)	4 (0.5%)	
	74	M	183 (81.3%)	106 (80.9%)	289 (81.2%)	1.000	217 (91.9%)	177 (95.7%)	221 (85.3%)	16 (10.2%)	631 (75.4%)	<**0.001**
		I	42 (18.7%)	25 (19.1%)	67 (18.8%)		19 (8.1%)	8 (4.3%)	38 (14.7%)	141 (89.8%)	206 (24.6%)	
	75	N	183 (81.3%)	106 (80.9%)	289 (81.2%)	1.000	217 (91.9%)	177 (95.7%)	221 (85.3%)	16 (10.2%)	631 (75.4%)	**<0.001**
		E	42 (18.7%)	25 (19.1%)	67 (18.8%)		19 (8.1%)	8 (4.3%)	38 (14.7%)	141 (89.8%)	206 (24.6%)	
	76	K	168 (74.7%)	105 (80.2%)	273 (76.7%)	0.246	214 (90.7%)	177 (95.7%)	216 (83.4%)	10 (6.4%)	617 (73.7%)	**<0.001**
		T	57 (25.3%)	26 (19.8%)	83 (23.3%)		22 (9.3%)	8 (4.3%)	43 (16.6%)	147 (93.6%)	220 (26.3%)	
	93	T	225 (100.0%)	131 (100.0%)	356 (100.0%)	—	236 (100.0%)	185 (100.0%)	259 (100.0%)	157 (100.0%)	837 (100.0%)	—
		S	0 (0.0%)	0 (0.0%)	0 (0.0%)		0 (0.0%)	0 (0.0%)	0 (0.0%)	0 (0.0%)	0 (0.0%)	
	97	H	225 (100.0%)	131 (100.0%)	356 (100.0%)	—	236 (100.0%)	185 (100.0%)	259 (100.0%)	157 (100.0%)	837 (100.0%)	—
		Y	0 (0.0%)	0 (0.0%)	0 (0.0%)		0 (0.0%)	0 (0.0%)	0 (0.0%)	0 (0.0%)	0 (0.0%)	
	145	F	225 (100.0%)	131 (100.0%)	356 (100.0%)	—	236 (100.0%)	185 (100.0%)	259 (100.0%)	157 (100.0%)	837 (100.0%)	—
		I	0 (0.0%)	0 (0.0%)	0 (0.0%)		0 (0.0%)	0 (0.0%)	0 (0.0%)	0 (0.0%)	0 (0.0%)	
	218	I	225 (100.0%)	131 (100.0%)	356 (100.0%)	—	236 (100.0%)	185 (100.0%)	259 (100.0%)	157 (100.0%)	837 (100.0%)	—
		F	0 (0.0%)	0 (0.0%)	0 (0.0%)		0 (0.0%)	0 (0.0%)	0 (0.0%)	0 (0.0%)	0 (0.0%)	
	343	M	225 (100.0%)	131 (100.0%)	356 (100.0%)	—	236 (100.0%)	185 (100.0%)	259 (100.0%)	157 (100.0%)	837 (100.0%)	—
		L	0 (0.0%)	0 (0.0%)	0 (0.0%)		0 (0.0%)	0 (0.0%)	0 (0.0%)	0 (0.0%)	0 (0.0%)	
	350	C	225 (100.0%)	131 (100.0%)	356 (100.0%)	—	236 (100.0%)	185 (100.0%)	259 (100.0%)	157 (100.0%)	837 (100.0%)	—
		R	0 (0.0%)	0 (0.0%)	0 (0.0%)		0 (0.0%)	0 (0.0%)	0 (0.0%)	0 (0.0%)	0 (0.0%)	
	353	G	225 (100.0%)	131 (100.0%)	356 (100.0%)	—	236 (100.0%)	185 (100.0%)	259 (100.0%)	157 (100.0%)	837 (100.0%)	—
		V	0 (0.0%)	0 (0.0%)	0 (0.0%)		0 (0.0%)	0 (0.0%)	0 (0.0%)	0 (0.0%)	0 (0.0%)	
	356	I	188 (83.6%)	129 (98.5%)	317 (89.0%)	**<0**.**001**	227 (96.2%)	185 (100.0%)	259 (100.0%)	155 (98.7%)	826 (98.7%)	**<0.001**
		T	37 (16.4%)	2 (1.5%)	39 (11.0%)		9 (3.8%)	0 (0.0%)	0 (0.0%)	2 (1.3%)	11 (1.3%)	
*Pfmdr1*	86	N	202 (90.2%)	131 (100.0%)	333 (93.8%)	**<0**.**001**	235 (99.6%)	183 (98.9%)	234 (90.3%)	90 (57.3%)	742 (88.6%)	**<0.001**
		Y	22 (9.8%)	0 (0.0%)	22 (6.2%)		1 (0.4%)	2 (1.1%)	24 (9.3%)	66 (42.0%)	93 (11.1%)	
	184	Y	54 (24.1%)	30 (22.9%)	84 (23.7%)	0.897	98 (41.5%)	144 (77.8%)	217 (83.8%)	123 (78.3%)	582 (69.5%)	**<0.001**
		F	170 (75.9%)	101 (77.1%)	271 (76.3%)		138 (58.5%)	41 (22.2%)	42 (16.2%)	34 (21.7%)	255 (30.5%)	
	1034	S	219 (97.8%)	131 (100.0%)	350 (98.6%)	0.162	235 (99.6%)	185 (100.0%)	259 (100.0%)	157 (100.0%)	836 (99.9%)	—
		C	5 (2.2%)	0 (0.0%)	5 (1.4%)		0 (0.0%)	0 (0.0%)	0 (0.0%)	0 (0.0%)	0 (0.0%)	
	1042	N	223 (99.6%)	131 (100.0%)	354 (99.7%)	1.000	236 (100.0%)	185 (100.0%)	259 (100.0%)	157 (100.0%)	837 (100.0%)	—
		D	1 (0.4%)	0 (0.0%)	1 (0.3%)		0 (0.0%)	0 (0.0%)	0 (0.0%)	0 (0.0%)	0 (0.0%)	
	1246	D	224 (100.0%)	130 (99.2%)	354 (99.7%)	0.369	236 (100.0%)	185 (100.0%)	259 (100.0%)	157 (100.0%)	837 (100.0%)	—
		Y	0 (0.0%)	1 (0.8%)	1 (0.3%)		0 (0.0%)	0 (0.0%)	0 (0.0%)	0 (0.0%)	0 (0.0%)	

Number and proportion of *P. falciparum* isolates successfully sequenced per gene and per site, and prevalence of non-synonymous mutations at codons of interest. Mixed wild-type/mutant infections are classified as mutant. Proportions are calculated relative to the number of isolates successfully sequenced for each gene. Codons 93, 97, 145, 218, 343, 350 and 353 of *Pfcrt* were monomorphic across all sites and are not shown. WT, wild-type.

### Polymorphisms in Pfkelch13

**Figure 2.**
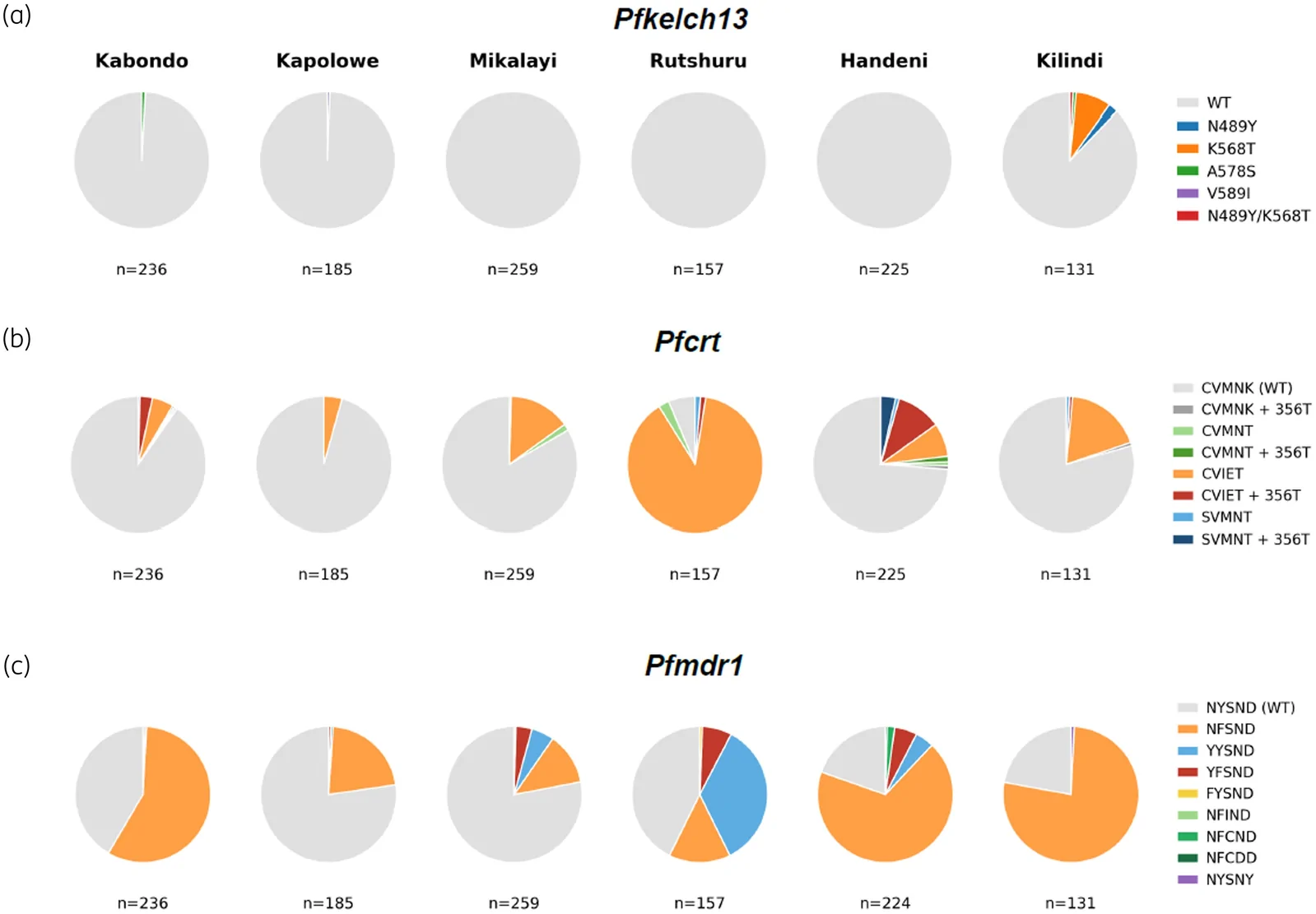
Site-level distribution of Pfkelch13, Pfcrt and Pfmdr1 polymorphisms in *P. falciparum* isolates from the DRC (Kabondo, Kapolowe, Mikalayi, Rutshuru) and Tanzania (Handeni, Kilindi). **(a)** Pfkelch13 allele frequencies per site (*n* = 1193). Wild-type (WT) in light grey; non-synonymous mutations N489Y, K568T, A578S, V589I, and the mixed N489Y/K568T allele detected in one Kilindi isolate, in colour. **(b)** Pfcrt haplotype frequencies (codons 72–76 and 356) per site (*n* = 1193). Three K76T-carrying haplotype backbones (CVIET, SVMNT, CVMNT) are shown with and without the additional 356 T substitution. **(c)** Pfmdr1 haplotype frequencies (codons 86, 184, 1034, 1042, 1246) per site (*n* = 1192). One Tanzanian isolate from Handeni with an incomplete haplotype was excluded. Sample size per site is indicated below each pie chart. WT, wild-type.

The wild-type allele predominated at 98.4% (1174/1193). Four non-synonymous mutations were detected at low frequency: K568T, N489Y, A578S and V589I (Figure 2a, Tables 1 and 2). None corresponded to validated or candidate ART-R markers. No validated mutations (R561H, P441L, C469Y) were detected.

**Table 2.** Distribution of *Pfcrt* (codons 72–76 and 356) and *Pfmdr1* (codons 86, 184, 1034, 1042 and 1246) haplotypes per study site, in Tanzania (2020–2021) and the Democratic Republic of the Congo (2017)

Gene	Allele	Haplotype	TANZANIA				DRC					
			Handeni	Kilindi	Total	*P*-value*	Kabondo	Kapolowe	Mikalayi	Rutshuru	Total	*P*-value*
No. of samples sequenced			225	131	356		236	185	259	157	837	
*Pfkelch13*	WT	—	225 (100.0%)	115 (87.8%)	340 (95.5%)	**<0**.**001**	234 (99.2%)	184 (99.5%)	259 (100.0%)	157 (100.0%)	834 (99.6%)	0.355
	N489Y	489 N > Y	0 (0.0%)	4 (3.1%)	4 (1.1%)	**0**.**018**	0 (0.0%)	0 (0.0%)	0 (0.0%)	0 (0.0%)	0 (0.0%)	—
	K568T	568 K > T	0 (0.0%)	12 (9.2%)	12 (3.4%)	**<0**.**001**	0 (0.0%)	0 (0.0%)	0 (0.0%)	0 (0.0%)	0 (0.0%)	—
	A578S	578 A > S	0 (0.0%)	1 (0.8%)	1 (0.3%)	0.368	2 (0.8%)	0 (0.0%)	0 (0.0%)	0 (0.0%)	2 (0.2%)	0.164
	V589I	589 V > I	0 (0.0%)	0 (0.0%)	0 (0.0%)	—	0 (0.0%)	1 (0.5%)	0 (0.0%)	0 (0.0%)	1 (0.1%)	0.317
*Pfcrt*	WT	CVMNKTHFIMCGI	166 (73.8%)	104 (79.4%)	270 (75.8%)	0.250	213 (90.3%)	177 (95.7%)	216 (83.4%)	10 (6.4%)	616 (73.6%)	**<0**.**001**
	76T	CVMN**T**THFIMCGI	2 (0.9%)	0 (0.0%)	2 (0.6%)	0.534	1 (0.4%)	0 (0.0%)	4 (1.5%)	4 (2.5%)	9 (1.1%)	0.083
	356T	CVMNKTHFIMCG**T**	2 (0.9%)	1 (0.8%)	3 (0.8%)	1.000	1 (0.4%)	0 (0.0%)	0 (0.0%)	0 (0.0%)	1 (0.1%)	0.466
	76 T/356T	CVMN**T**THFIMCG**T**	3 (1.3%)	0 (0.0%)	3 (0.8%)	0.300	1 (0.4%)	0 (0.0%)	0 (0.0%)	0 (0.0%)	1 (0.1%)	0.466
	72S/76T	**S**VMN**T**THFIMCGI	2 (0.9%)	1 (0.8%)	3 (0.8%)	1.000	1 (0.4%)	0 (0.0%)	1 (0.4%)	2 (1.3%)	4 (0.5%)	0.385
	74I/75E/76T	CV**IET**THFIMCGI	18 (8.0%)	24 (18.3%)	42 (11.8%)	0.006	12 (5.1%)	8 (4.3%)	38 (14.7%)	139 (88.5%)	197 (23.5%)	**<0**.**001**
	72S/76 T/356T	**S**VMN**T**THFIMCG**T**	8 (3.6%)	0 (0.0%)	8 (2.2%)	**0**.**029**	0 (0.0%)	0 (0.0%)	0 (0.0%)	0 (0.0%)	0 (0.0%)	—
	74I/75E/76 T/356T	CV**IET**THFIMCG**T**	24 (10.7%)	1 (0.8%)	25 (7.0%)	**<0**.**001**	7 (3.0%)	0 (0.0%)	0 (0.0%)	2 (1.3%)	9 (1.1%)	**0**.**005**
*Pfmdr1*	WT	NYSND	44 (19.6%)	29 (22.1%)	73 (20.5%)	0.587	98 (41.5%)	143 (77.3%)	202 (78.0%)	67 (42.7%)	510 (60.9%)	**<0**.**001**
	86Y	**Y**YSND	10 (4.4%)	0 (0.0%)	10 (2.8%)	**0**.**016**	0 (0.0%)	1 (0.5%)	14 (5.4%)	55 (35.0%)	70 (8.4%)	**<0**.**001**
	86F	**F**YSND	0 (0.0%)	0 (0.0%)	0 (0.0%)	—	0 (0.0%)	0 (0.0%)	1 (0.4%)	1 (0.6%)	2 (0.2%)	0.515
	184F	N**F**SND	153 (68.0%)	101 (77.1%)	254 (71.3%)	0.070	136 (57.6%)	40 (21.6%)	32 (12.4%)	23 (14.6%)	231 (27.6%)	**<0**.**001**
	1246Y	NYSN**Y**	0 (0.0%)	1 (0.8%)	1 (0.3%)	0.368	0 (0.0%)	0 (0.0%)	0 (0.0%)	0 (0.0%)	0 (0.0%)	—
	86Y/184F	**YF**SND	12 (5.3%)	0 (0.0%)	12 (3.4%)	**0**.**005**	1 (0.4%)	1 (0.5%)	10 (3.9%)	11 (7.0%)	23 (2.7%)	**<0**.**001**
	184F/1034I	N**FI**ND	0 (0.0%)	0 (0.0%)	0 (0.0%)	—	1 (0.4%)	0 (0.0%)	0 (0.0%)	0 (0.0%)	1 (0.1%)	0.466
	184F/1034C	N**FC**ND	4 (1.8%)	0 (0.0%)	4 (1.1%)	0.301	0 (0.0%)	0 (0.0%)	0 (0.0%)	0 (0.0%)	0 (0.0%)	—
	184F/1034C/1042D	N**FCD**D	1 (0.4%)	0 (0.0%)	1 (0.3%)	1.000	0 (0.0%)	0 (0.0%)	0 (0.0%)	0 (0.0%)	0 (0.0%)	—

Multi-codon haplotypes were inferred directly from sequencing reads. Isolates with missing data at one or more codons of a given haplotype were excluded from the corresponding haplotype analysis (one *Pfmdr1* isolate from Handeni excluded; *n* = 224 instead of 225). Mixed wild-type/mutant infections at any codon are classified as mutant for haplotype assignment. Bold proportions in the *Pfcrt* column indicate haplotypes carrying the K76T mutation. *Pfcrt* nomenclature: codons 72–73–74–75–76 + 93–97–145–218–343–350–353–356. WT, wild-type.

All Tanzanian mutants were from Kilindi (12.2%), while none were detected in Handeni (Fisher’s exact test, *P* < 0.001). Whithin Kilindi, K568T was most frequent (9.2%), followed by N489Y (3.1%) and A578S (0.8%), with one mixed allele. In the DRC, three mutants were observed: A578S in Kabondo (0.8%) and V589I in Kapolowe (0.5%), with none in Mikalayi or Rutshuru.

### Polymorphisms in *pfcrt*

*Codon-level frequencies.* All 1193 isolates were analysed (Tables 1 and 2). The K76T prevalence was 23.3% in Tanzanian and 26.3% in the DRC isolates. M74I and N75E showed identical frequencies (Tanzania 18.8% and DRC 24.6%), consistent with CVIET linkage. The 72S mutation was rare (3.1% versus 0.5%). The 356 T variant occurred in 11.0% (Tanzanian) and 1.3% (DRC) isolates. All other codons were monomorphic. In Tanzania, only 356 T differed significantly between sites (16.4% versus 1.5%, χ2 test, *P* < 0.001). In the DRC, K76T showed significant heterogeneity across sites (χ^2^ test, *P* < 0.001), driven by high prevalence in Rutshuru.

*Haplotype distribution*. The wild-type CVMNKTHFIMCGI haplotype predominated in both countries (75.8% in Tanzania and 73.6% in DRC) (Figure 2b). Three K76T-based haplotypes were identified (CV**IET**, **S**VMNT and CVMN**T)**, each with or without 356 T substitution. The definition of *Pfcrt* haplotypes according to the analysed codon positions is provided in **Table S1** (available as Supplementary data at *JAC* Online). A minor haplotype carrying 356 T alone was also observed.

In Tanzania, CV**IET**THFIMCGI (11.8%) and CV**IET**THFIMCG**T** (7.0%) predominated among mutants, the latter concentrated in Handeni (10.7% versus 0.8%, Fisher’s exact test, *P* < 0.001). **S**VMN**T**THFIMCG**T** was restricted to Handeni (Fisher’s exact test, *P* = 0.029).

In the DRC, CV**IET**THFIMCGI dominated (23.5%), with significant clustering in Rutshuru (88.5%, Fisher’s exact test, *P* < 0.001). CV**IET**THFIMCG**T** remained rare (1.1%), mainly in Kabondo (3.0%).

The candidate 7G8 haplotype could not be assessed, as several codons were not included in the amplicon design.

### Polymorphisms in Pfmdr1

A total of 1193 isolates were analysed (DRC *n* = 837, Tanzania *n* = 356). One Tanzanian isolate with incomplete data was excluded from haplotype analysis (Tables 1 and 2).

*Codon-level frequencies.* The 86Y mutation occurred in 6.2% (Tanzania) and 11.1% (DRC). The 184F variant was frequent in Tanzania (76.1%) but lower in the DRC (30.5%). Mutations at codons 1034, 1042 and 1246 were rare (≤1.4%), with 1034C absent in the DRC.

In Tanzania, 86Y was confined to Handeni (9.8%, Fisher’s exact test, *P* < 0.001), while 184F was similar across sites. In the DRC, 86Y ranged from 0.4% (Kabondo) to 42.0% (Rutshuru), and 184F also varied significantly across sites (58.5% at Kabondo versus 16.2%–22.2% elsewhere, χ^2^ test, *P* < 0.001).

*Haplotype distribution* (Figure 2c). The definition of *Pfmdr* haplotypes according to the analysed codon positions is provided in **Table S1**. In Tanzania, N**F**SND predominated at 71.5% (254/355), followed by NYSND (20.6%), **YF**SND (3.4%) and **Y**YSND (2.8%). Rare haplotypes were infrequent. **YF**SND and **Y**YSND were restricted to Handeni (5.3% and 4.4%), consistent with the 86Y distribution. In the DRC, NYSND was most frequent (60.9%), followed by N**F**SND (27.6%), **Y**YSND (8.4%) and **YF**SND (2.7%). All haplotypes differed significantly between sites (χ^2^ test, *P* < 0.001), defining three patterns: NYSND-dominance (Kapolowe and Mikalayi), N**F**SND-enrichment (Kabondo), and 86Y-associated in Rutshuru, where **Y**YSND reached 35.0%, and the combined 86Y-bearing haplotypes reached 42.0%.

## Discussion

This study analysed baseline molecular markers of artemisinin and partner drug resistance in *P. falciparum* isolates from the DRC (2017) and Tanzania (2020–2021), two malaria-endemic countries with different epidemiological trajectories. Tanzania is among the East African countries where ART-R has been documented.^10–12^ The DRC, where ART-R had not been reported at the time of sample collection,^24^ shares borders with antimalarial drug resistance hotspots and was therefore a priority for early molecular surveillance.^25^ The 2017 TES *P. falciparum* isolates from the DRC had previously raised concerns regarding AL efficacy in some provinces, following evidence of efficacy below 90% at the Mikalayi site, located near Angola’s Lunda province, where reduced AL efficacy had also been reported. This TES study characterized *Pfkelch13*, *Pfcrt*, and *Pfmdr1* polymorphisms using Sanger sequencing,^21^ which has limited sensitivity for minority variants. The present study complements those findings through targeted deep amplicon sequencing and a harmonized workflow, providing higher-resolution insights into antimalarial resistance markers in the DRC.

To place these findings into a broader regional context, we included molecular data from Tanzania, one of the African hotspots for antimalarial drug resistance. Similarly, the Tanzanian *P. falciparum* isolates analysed in this study were collected during a previously published trial assessing the feasibility and effectiveness of IPTsc using dihydroartemisinin-piperaquine against standard of care.^22^ Although the original study focused on clinical and epidemiological outcomes, molecular analyses of resistance-associated parasite genes were not previously performed.^22^ By leveraging these archived samples, the present study extends the value of this biobank and establishes a valuable molecular baseline for key antimalarial resistance markers. When considered together, the data generated from both the DRC and Tanzania provide an important regional reference framework for monitoring the emergence and spread of molecular markers associated with artemisinin and partner drug resistance in *P. falciparum* populations across malaria-endemic regions of SSA.

Although no validated *Pfkelch13* marker was detected in either country, the partner drug profiles differed substantially between the two countries. The wild-type *Pfkelch13* allele predominated in both countries (98.4%). Four non-synonymous mutations were detected at low frequency (N489Y, K568T, A578S and V589I), none classified as a validated, candidate or potential marker of ART-R.^13,14^ The validated markers reported in neighbouring East African settings (R561H, R622I, C469Y, A675 V) were absent. These inter-country differences may reflect contrasting malaria transmission dynamics, antimalarial drug pressure and regional parasite connectivity.^26^ Tanzania has undergone prolonged ACT use and is located within the East African corridor where *Pfkelch13*-mediated ART-R has increasingly been documented, whereas the DRC historically remained outside the main resistance epicentres despite bordering Rwanda and Uganda. Differences in transmission intensity may also influence the emergence and dissemination of resistant lineages, since high-transmission settings can reduce selective sweeps through greater parasite recombination. In eastern DRC, substantial population mobility and cross-border exchange with neighbouring ART-R hotspots may nevertheless facilitate parasite flow and the introduction of resistant variants. This regional connectivity is supported by the subsequent detection of the validated *Pfkelch13* mutations R561H in Kabondo sentinel site (Northern Tshopo Province), candidate P441L mutation in sentinel site Rutshuru (North Kivu province),^19^ and more recently the detection of the validated *Pfkelch13* mutations A675 V and C469Y in northeastern Ituri province which borders South Sudan to the north and Uganda to the east,^26^ highlighting the epidemiological linkage between eastern DRC and the broader East African ART-R corridor.

All *Pfkelch13* mutant isolates identified in Tanzania were located at Kilindi (16/131, 12.2%), with no mutation detected at Handeni, a localized mutation cluster that should be interpreted cautiously given the absence of phenotypic data and the small site-level numbers. The A578S substitution has been identified in Africa but is not currently classified as an artemisinin resistance marker, as no association with clinical or *in vitro* ART-R has been demonstrated.^27,28^ The 2017 DRC baseline reported here no longer reflects the current situation. R561H and P441L have been confirmed in the same DRC sentinel sites in 2021,^19^ while C469Y and A675 V have also been detected in South Kivu and Ituri provinces, which directly border East African ART-R hotspots.^20,26^ This trajectory underscores the need to integrate enhanced genomic surveillance into routine TES and to prepare contingency strategies before validated markers spread further.

The K76T mutation, a validated marker of CQ resistance,^15,16^ was detected at comparable overall frequencies in Tanzania (23.3%) and the DRC (26.3%), but with marked heterogeneity between DRC sentinel sites (4.3% at Kabondo to 93.6% at Rutshuru). The persistence of K76T more than two decades after CQ withdrawal is consistent with reports across SSA^29–31^ and likely reflects the residual fitness of mutant parasites combined with continued informal CQ use, particularly through private outlets in the DRC.^32^ Although the chemical similarity between CQ and AQ has historically been invoked to explain K76T maintenance under AQ pressure, the WHO compendium currently lists K76T and the CV**IET** haplotype as validated markers of CQ resistance only. CV**IET** dominated the mutant *Pfcrt* profile and accounted for much of the inter-site heterogeneity observed in the DRC, reaching 88.5% of isolates in Rutshuru. This site also showed the highest proportion of 86Y-carrying *Pfmdr1* haplotypes (42%) and the lowest N**F**SND proportion, a combination consistent with sustained 4-aminoquinoline pressure and limited lumefantrine exposure. Two non-exclusive factors may contribute. First, historical persistence may contribute, as K76T was already reported at 93% in eastern DRC in the early 2000s,^33^ and mutant haplotypes may persist after chloroquine withdrawal. Second, although AS-AQ and AL are recommended nationwide, effective drug pressure may differ in conflict-affected Rutshuru, where disrupted health services may favour self-medication with non-recommended antimalarials, including chloroquine. A comparable *Pfcrt* profile has been reported in Katana, another conflict-affected site in eastern DRC.^34^

The CV**IET** haplotype carrying the additional 356 T substitution, present in 7.0% of Tanzanian isolates and almost confined to Handeni, may correspond to a regional sub-lineage. The **S**VMN**T** haplotype, classified as a candidate marker of CQ-R,^35^ was detected at low frequency in both countries (3.1% in Tanzania, 0.5% in the DRC). The **S**VMN**T**THFIMCG**T** variant was confined to Handeni (8/225, 3.6%), a configuration reported only sporadically in East Africa.^36^ The WHO compendium recognizes only one *Pfcrt* haplotype (7G8: C72S + K76T + A220S + N326D + I356L) as a candidate marker of AQ-R;^17^ codons 220, 326 and 371 were not covered by our amplicon design, precluding direct assessment of this marker.

The most striking divergence between the two countries was observed for *Pfmdr1*. In Tanzania, the N**F**SND haplotype predominated (71.5%), while in the DRC the wild-type NYSND remained the most frequent (60.9%), with N**F**SND found in only 27.6% of isolates. The 184F variant followed the same pattern (76.1% in Tanzania, 30.5% in the DRC), whereas 86Y was twice as frequent in the DRC (11.1%) as in Tanzania (6.2%). These haplotype distributions are consistent with the differential drug pressure exerted by the dominant first-line ACT in each country: AL in Tanzania, and combined AS-AQ/AL deployment in the DRC.^37^ The WHO compendium does not currently classify N**F**SND as a validated or candidate marker of lumefantrine resistance: although N86 and 184F alleles have shown modestly reduced *in vitro* lumefantrine susceptibility, the difference is limited and inconsistent across populations, and these alleles are described as a signal of selection rather than evidence of resistance.^14^ The high N**F**SND prevalence in Tanzania likely reflects sustained AL selection rather than clinical lumefantrine resistance. Notably, *Pfmdr1* gene amplification is currently the only lumefantrine resistance marker listed by the WHO compendium, and is classified as a potential marker.^14,18^ However, this copy number variation is not assessable by amplicon sequencing.^18^ The 86Y mutation, by contrast, is a candidate marker of AQ-R,^14^ and its higher prevalence in the DRC is consistent with the long-standing use of AS-AQ, although residual CQ pressure may also contribute. Within the DRC, 86Y showed marked variation between sites (0.4% at Kabondo to 42.0% at Rutshuru), revealing three distinct *Pfmdr1* profiles: N**F**SND-enriched at Kabondo, NYSND-dominated at Kapolowe and Mikalayi, and 86Y-enriched at Rutshuru where **Y**YSND alone reached 35.0%. This intra-country heterogeneity points to different local selection patterns, possibly reflecting differences in dominant ACT use, residual CQ pressure, or parasite population structure between sentinel sites.

The current DRC policy of maintaining two first-line ACTs (AS-AQ and AL) is supported by modelling evidence that multiple first-line therapies (MFT) deployment reduces drug pressure on any single combination and delays the fixation of resistant parasites. The contrasting partner-drug signatures observed across DRC sites in our data, N**F**SND-enriched at Kabondo, 86Y-enriched at Rutshuru, show that an MFT environment generates locally distinct selection profiles, and reinforce the rationale for a coordinated MFT strategy informed by molecular and clinical surveillance. The phased introduction of newly available combinations such as artesunate-pyronaridine or artesunate-mefloquine, and ultimately the deployment of triple artemisinin-based combination therapies (TACTs), should be considered if the efficacy of current ACTs deteriorates.^3^

Several limitations should be acknowledged. First, the DRC and Tanzanian samples were collected at different time points (2017 and 2020–2021), age groups and clinical contexts (TES enrolling young children with uncomplicated malaria in the DRC; IPTsc trial enrolling schoolchildren in Tanzania), which precludes direct inter-country statistical comparison and limits causal interpretation. Second, the amplicon design did not cover *Pfcrt* codons 220, 326 and 371 needed to identify the 7G8 candidate AQ-R haplotype and did not allow detection of *Pfmdr1* gene amplification. Third, the DRC baseline reported here predates the documented emergence of validated *Pfkelch13* mutations in the country and should be considered together with more recent surveillance data. However, this study provides an early reference point for subsequent surveillance. The absence of validated *Pfkelch13* ART-R markers in these samples is consistent with the continued efficacy of ACTs at the time of sampling but should be interpreted alongside contemporary TES. The near-fixation of the CV**IET** haplotype in Rutshuru (∼89%) shows strong CQ and related analogues drug pressure and calls for intensified molecular surveillance as well as regular TES on the border areas where parasite movement may influence the spread of resistance-associated lineages.

### Conclusion

This study reports baseline prevalences of *P. falciparum* antimalarial resistance markers in the DRC (2017) and Tanzania (2020–2021), representing parasite populations before the subsequent detection of ART-R associated markers in these settings. No validated *Pfkelch13* marker was detected at any of the study sites, although our DRC baseline has since been overtaken by reports of R561H, P441L, A675 V and C469Y in the same and neighbouring provinces. These findings highlight the importance of sustained molecular surveillance integrated with TES to monitor the evolution of resistance and inform future malaria control strategies.

## Supplementary Material

dkag309_Supplementary_Data

## Data Availability

The aggregated genotyping data supporting the findings of this study are presented within the article and its tables. Individual-level sequencing data and patient metadata are available from the corresponding author upon reasonable request, subject to the ethical and data protection requirements of the participating institutions in the Democratic Republic of the Congo and Tanzania.

## Supplementary data

Table S1 is available as Supplementary data at *JAC* Online.
