# Supplementary material for: Genomic profiling of drug resistance markers in Plasmodium falciparum samples from the Democratic Republic of the Congo (2017) and Tanzania (2020–2021)
Source: J Antimicrob Chemother. 2026 Sep 1;81(10):dkag309. doi: 10.1093/jac/dkag309 (PMC13531235; doi:10.1093/jac/dkag309)
Supplement: Supplementary file 1 [file dkag309_supplementary_data.docx]

**Supplementary Table 1**. Definition of Pfcrt and Pfmdr1 haplotypes according to analyzed codon positions

| **Gene** | **Analyzed codon positions** | **Haplotype designation** | **Amino acid composition** |
| --- | --- | --- | --- |
| *Pfcrt* | 72–76, 356 | CVMNKTHFIMCGI | C72–V73–M74–N75–K76–I356 |
| *Pfcrt* | 72–76, 356 | CVIETTHFIMCGI | C72–V73–I74–E75–T76–I356 |
| *Pfcrt* | 72–76, 356 | CVIETTHFIMCGT | C72–V73–I74–E75–T76–T356 |
| *Pfcrt* | 72–76, 356 | SVMNTTHFIMCGT | S72–V73–M74–N75–T76–T356 |
| *Pfcrt* | 72–76, 356 | CVMNTTHFIMCGI | C72–V73–M74–N75–T76–I356 |
| *Pfcrt* | 72–76, 356 | CVMNKTHFIMCGT | C72–V73–M74–N75–K76–T356 |
| *Pfmdr1* | 86, 184, 1034, 1042, 1246 | NFSND | N86–F184–S1034–N1042–D1246 |
| *Pfmdr1* | 86, 184, 1034, 1042, 1246 | NYSND | N86–Y184–S1034–N1042–D1246 |
| *Pfmdr1* | 86, 184, 1034, 1042, 1246 | YFSND | Y86–F184–S1034–N1042–D1246 |
| *Pfmdr1* | 86, 184, 1034, 1042, 1246 | YYSND | Y86–Y184–S1034–N1042–D1246 |

Haplotypes were defined according to the amino acid residues at the analyzed codon positions. Frequencies and geographical distribution of haplotypes are presented in the main text and **Tables 1–2.**
